# Knowledge mapping of autophagy in osteoarthritis from 2004 to 2022: A bibliometric analysis

**DOI:** 10.3389/fimmu.2023.1063018

**Published:** 2023-03-09

**Authors:** Jiahe Liao, Xinbo Yu, Jiaqi Chen, Zihua Wu, Qian He, Yan Zhang, Weijiang Song, Jing Luo, Qingwen Tao

**Affiliations:** ^1^ Graduate School, Beijing University of Chinese Medicine, Beijing, China; ^2^ Traditional Chinese Medicine Department of Rheumatism, China-Japan Friendship Hospital, Beijing, China; ^3^ Traditional Chinese Medicine Department, Peking University Third Hospital, Beijing, China; ^4^ Beijing Key Laboratory of Immune Inflammatory Disease, China-Japan Friendship Hospital, Beijing, China

**Keywords:** osteoarthritis, autophagy, bibliometrics, research hotspot, data visualization

## Abstract

**Background:**

Autophagy in osteoarthritis (OA) has become an active area of research with substantial value and potential. Nevertheless, few bibliometric studies have systematically analyzed the available research in the field. The main goal of this study was to map the available literature on the role of autophagy in OA and identify global research hotspots and trends.

**Methods:**

The Web of Science Core Collection and Scopus databases were interrogated for studies of autophagy in OA published between 2004 and 2022. Microsoft Excel, VOSviewer and CiteSpace software were used to analyze and visualize the number of publications and associated citations, and reveal global research hotspots and trends in the autophagy in OA field.

**Results:**

732 outputs published by 329 institutions from 55 countries/regions were included in this study. From 2004 to 2022, the number of publications increased. China produced the most publications (n=456), prior to the USA (n=115), South Korea (n=33), and Japan (n=27). Scripps Research Institute (n=26) was the most productive institution. Martin Lotz (n=30) was the highest output author, while Caramés B (n=302) was the highest output author. *Osteoarthritis and Cartilage* was the most prolific and most co-cited journal. Currently, the autophagy in OA research hotspots include chondrocyte, transforming growth factor beta 1 (TGF-β1), inflammatory response, stress, and mitophagy. The emerging research trends in this field are AMPK, macrophage, senescence, apoptosis, tougu xiaotong capsule (TXC), green tea extract, rapamycin, and dexamethasone. Novel drugs targeting specific molecule such as TGF-β and AMPK have shown therapeutic potential but are still in the preclinical stage of development.

**Conclusions:**

Research on the role of autophagy in OA is flourishing. Martin Lotz, Beatriz Caramés, and *Osteoarthritis and Cartilage* have made outstanding contributions to the field. Prior studies of OA autophagy mainly focused on mechanisms underlying OA and autophagy, including AMPK, macrophages, TGF-β1, inflammatory response, stress, and mitophagy. Emerging research trends, however, are centered around the relationship between autophagy, apoptosis, and senescence, as well as drug candidates such as TXC and green tea extract. The development of new targeted drugs that enhance or restore autophagic activity is a promising strategy for the treatment of OA.

## 1 Introduction

Osteoarthritis (OA) is a common chronic degenerative joint disorder, which affects heavily manipulated or loaded joints and is often accompanied by structural damage and dysfunction such as joint pain, stiffness, hypertrophy, deformity, and restricted movement (1). OA is characterized by the degradation of the articular cartilage and concomitant adaptive osteogenesis (2). Guidelines of OA in China (2019 edition) divided OA into primary and secondary according to the etiology (3). At present, the cause of primary OA is not clear. Age, obesity, genetics, sex, and joint biomechanics are considered risk factors for OA (4). Inflammation at joint sites may cause inflammation-based OA, while the disorder of bone metabolism or changes in the metabolic environment of joints may result in metabolic-based OA (5). Aging, obesity, and the frequency of joint injuries are on the rise. Consequently, the prevalence of OA has also increased, with a worldwide estimate of 250 million people are currently affected (6). In older adults, OA has gradually become the leading cause of disability and reduced quality of life. In addition, OA poses a great economic burden on society. At present, the main forms of treatment for OA include physiotherapy, non-steroidal anti-inflammatory drugs (NSAIDs), analgesics, corticosteroids, and eventual arthroplasty (7). There is still no targeted and effective disease-modifying drug for OA. Thus, there is an urgent need to elucidate the pathogenesis of OA to develop new effective and safe therapeutic strategies. A recent report suggests that the pathogenesis of OA may be closely related to inflammation, oxidative stress, apoptosis, and autophagy (8). The study of autophagy in the context of OA pathogenesis has become an active research area with promising potential.

Autophagy is a cellular degradation and recycling process highly conserved in all eukaryotes, and is particularly important in cartilage repair (9). Autophagy plays an important role in maintaining cellular homeostasis by mediating both cell survival and apoptosis through complex steps (10). Autophagy is involved not only in aging but also in diseases with inflammatory components, such as OA, metabolic disorders, neurodegeneration, infections, cancer, and autoimmune, cardiovascular, and hepatic diseases (11). In chondrocytes, autophagy acts as a protective or survival mechanism, and can be triggered by various stressors (e.g., nutrient deprivation, oxidative stress, inflammatory response, and pathogens) (12). Recent studies have established the important role of autophagy in regulating the pathogenesis of OA and OA-related gene expression in chondrocytes (13, 14). Promoting and maintaining appropriate levels of autophagy in chondrocytes has been considered as new potential therapeutic target.

Bibliometrics uses mathematical and statistical methods to study published research documents and the associated bibliometric characteristics, such as countries/regions, institutions, journals, authors, and citations (15). To date, no bibliometric study of OA autophagy has been published to inform the quantitative relationships, distribution structure, and changing patterns of research in this field. Thus, in this study, we aimed to explore publications on autophagy in OA from 2004 to 2022, and reveal the global research hotspots and trends.

## 2 Materials and methods

### 2.1 Data collection

Web of Science Core Collection (WoSCC) and Scopus are the main sources of citation data, with Scopus offering a larger journal coverage in all fields. The interdisciplinary coverage provided by these databases is highly valuable for the study and comparison of different scientific fields especially in the Natural Sciences, Engineering, and Biomedical Research domains (16). Therefore, the data for this study were obtained from both databases.

**Figure 1:**
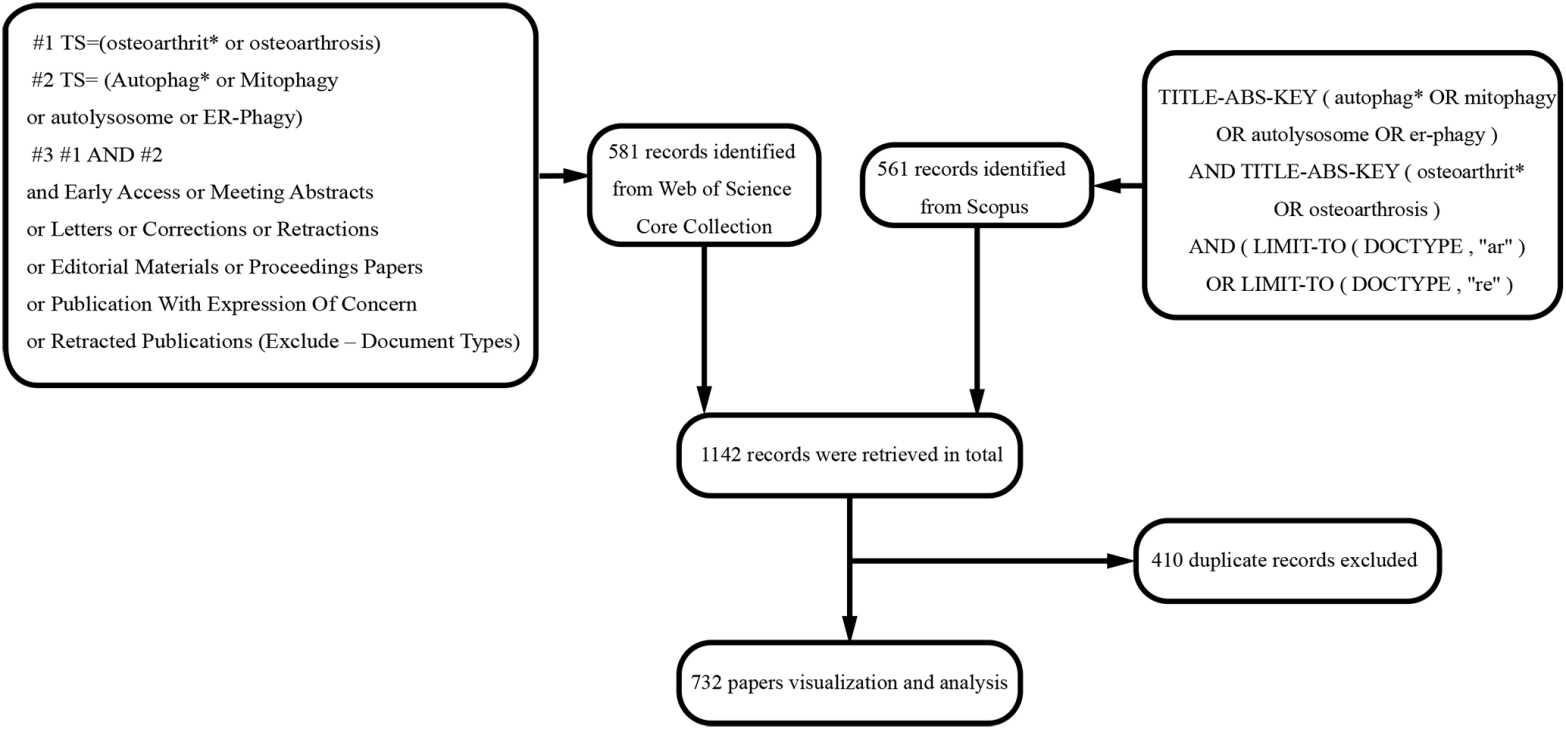
Flowchart of literature search and selection.

We searched WoSCC and Scopus from 2004 to June 23, 2022. The data search strategy included the topic “autophag* or mitophagy or autolysosome or er-phagy” and the topic “osteoarthrit* or osteoarthrosis”. Early access, meeting abstracts, letters, corrections, retractions, editorial materials, proceedings, publication with expression of concern, and retracted publications were excluded. Original and review articles reporting autophagy in OA were qualified for inclusion regardless of language. Our search identified 1142 records fulfilling the inclusion criteria: 581 papers from WoSCC and 561 from Scopus. After further screening the titles, abstracts, and full texts, 410 duplicates were excluded. Finally, 732 papers were included in this study. The search and selection process is shown in **Figure 1**.

### 2.2 Data analysis

All included papers retrieved from WoSCC and Scopus were imported into Microsoft Excel, VOSviewer, and CiteSpace software for analyzing and visualizing. Microsoft Excel 2019 (Microsoft Corp, Redmond, Washington USA) was used to evaluate the number of papers published in each year and journal. CiteSpace is an interactive visualization tool, which combines information visualization methods, bibliometrics, and data mining algorithms to extract patterns from citation data (17). CiteSpace (V.6.1.R2) software (Chaomei Chen, Philadelphia, Pennsylvania, USA) was used to conduct visual analysis of the distribution of authors, institutions, countries/regions, keyword clusters, co-cited references, co-cited authors, co-cited journals, and timelines. VOSviewer (V.1.6.18) software (Leiden University, Leiden, the Netherlands) was used to provide density visualization.

## 3 Results

### 3.1 The publication outputs trend

**Figure 2:**
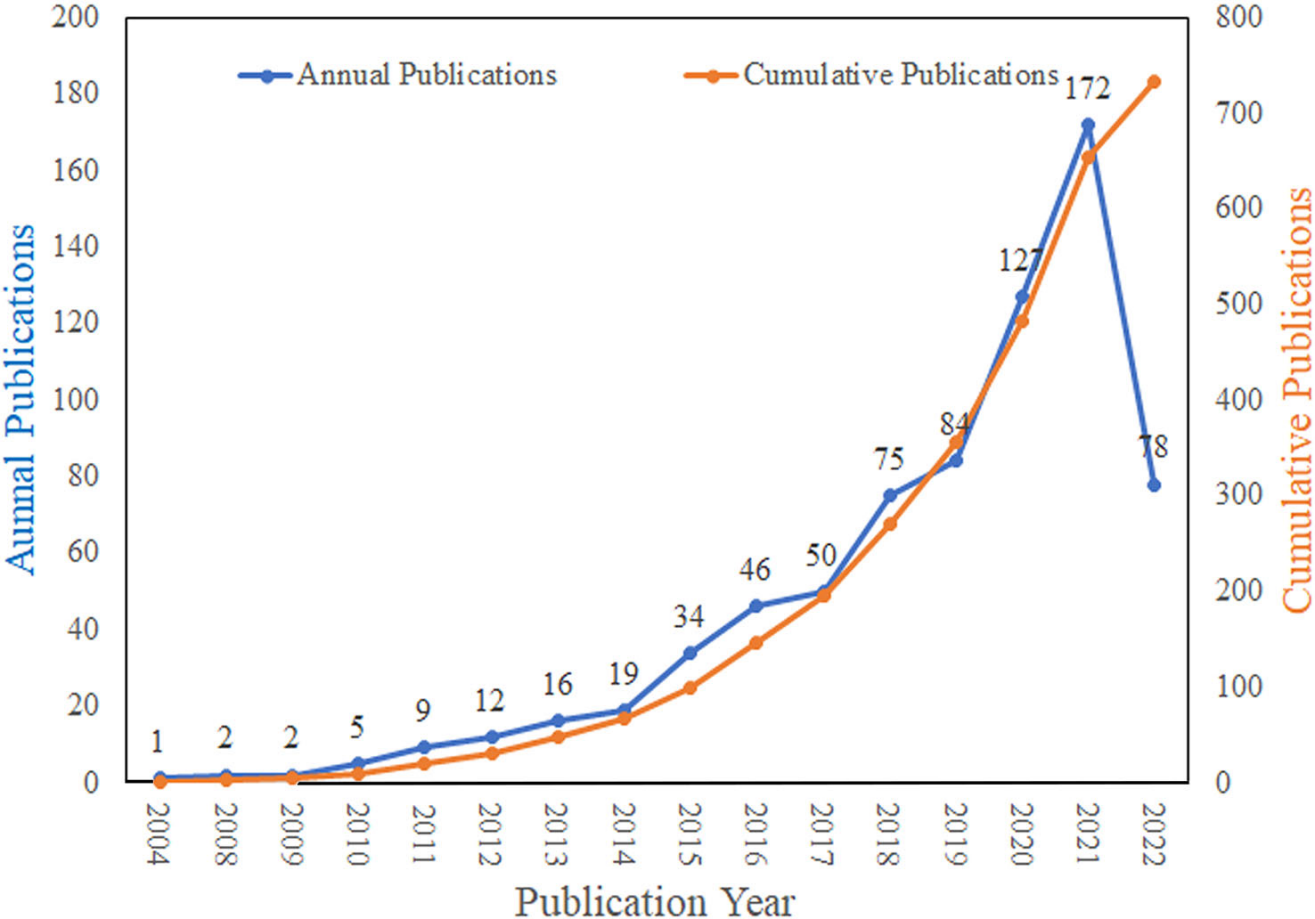
Trends of publications on autophagy in OA from 2004 to 2022.

A total of 732 papers (626 original and 87 review articles) published between May 2004 and June 2022 were included in this study. General information relating to the data used in this study is presented in **Table 1**. From 2004 to 2022, the cumulative number of publications on autophagy in OA increased year by year (**Figure 2**). From 2009 to 2021, the annual number publications increased steadily and reached its maximum in 2021, with a total of 172 outputs (23.50%). Between January and June 2022, the number of outputs reached 78 (10.66%), which was similar to the whole outputs in 2019. These trends indicate that autophagy in OA research is flourishing.

**Table 1:** General information on papers related to autophagy in OA published from 2004 to 2022.

Articles	732
Citations	3461
Average citations per articles	7.80
Authors	444
Articles per author	1.65
Articles per Coauthor	1.09
Sources (journals)	160
Keywords	508

### 3.2 Distribution of countries/regions and institutions

732 outputs published by 329 institutions from 55 countries/regions were included in this study. As shown in **Table 2**, China produced the most publications (n=456, 62.30%), prior to the USA (n=115, 15.71%), South Korea (n=33, 4.51%), Japan (n=27, 3.69%), and Spain (n=25, 3.42%). Scripps Research Institute was the most productive publishing institution (n=26, 3.55%), followed by Zhejiang University (n=23, 3.14%), and Shanghai Jiao Tong University (n=22, 3.01%). Wenzhou Medical University, Nanjing Medical University, and China Medical University each had the same number of outputs (n=19, 2.60%).

**Table 2:** Distribution of publications on autophagy in OA from different countries/regions and institutions.

Rank	Country/Region	Year	Centrality	Count (%)	Institution	Year	Centrality	Count (%)
1	CHINA	2012	0.53	456 (62.30)	Scripps Res Inst	2010	0.09	26 (3.55)
2	USA	2008	0.35	115 (15.71)	Zhejiang Univ	2013	0.02	23 (3.14)
3	SOUTH KOREA	2010	0.04	33 (4.51)	Shanghai Jiao Tong Univ	2014	0.01	22 (3.01)
4	JAPAN	2012	0.08	27 (3.69)	Wenzhou Med Univ	2017	0.01	19 (2.60)
5	SPAIN	2009	0.30	25 (3.42)	Nanjing Med Univ	2014	0.00	19 (2.60)
6	ITALY	2012	0.07	23 (3.14)	China Med Univ	2017	0.03	19 (2.60)
7	ENGLAND	2004	0.18	19 (2.60)	Huazhong Univ Sci & Technol	2013	0.02	18 (2.46)
8	NETHERLANDS	2011	0.02	15 (2.05)	Cent South Univ	2020	0.00	13 (1.78)
9	CANADA	2015	0.01	14 (1.91)	Fourth Mil Med Univ	2013	0.01	13 (1.78)
10	GERMANY	2004	0.08	14 (1.91)	Anhui Med Univ	2013	0.00	12 (1.64)

**Figure 3:**
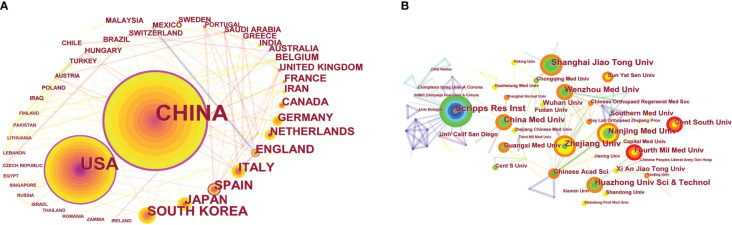
CiteSpace visualization map of publications on autophagy in OA from different countries/regions **(A)** and institutions **(B)**.

**Figure 3** shows the visual map of publications related to autophagy in the field of OA originating from different countries/regions and institutions. Each node represents a country or institution, and the size of the node is proportional to the number of outputs. Similarly, the number of citations is related to the size of each node’s label. The lines between the circles denote collaboration between countries or institutions; the wider the line, the stronger the collaboration. The centrality of nodes in the knowledge map is considered as a measure of the node significance and represents the interaction between nodes. Nodes with high centrality are outlined with a purple circle. In addition, the circles inside the nodes represent the number of publications produced by a country or institution in a given year, with different colors representing different years.

As shown in **Figure 3A**, the deeper the ring color, the lower the publication year; and the lighter the ring color, the more recent the publication. China ranked first in terms of the volume of recent publications on autophagy in OA. Papers published by the USA originated from the 2008–2022 period, while China published most of its outputs after 2012. Five nodes with high centrality, namely China (centrality=0.53), the USA (centrality=0.35), Spain (centrality=0.30), England (centrality=0.18), and Saudi Arabia (centrality=0.18) were identified. Thus, these countries are the major collaborators in the field of OA autophagy.

As shown in **Figure 3B**, colors vary from grey to red as time goes from 2010 to 2022. Scripps Research Institute was the most productive institution, but its centrality was relatively low (centrality=0.09). All the top 10 publishing institutions had low centrality.

### 3.3 Authors and co-cited authors

In total, 444 authors have made contributions to this field. As shown in **Table S1**, Martin Lotz had the most significant number of publications on autophagy in OA (n=30, 4.10%), ahead of Beatriz Caramés (n=13, 1.78%), and Bai Lunhao (n=10, 1.37%). It is worth noting that the centrality of authors was relatively low (≤0.01), suggesting a lack of influential authors in the field of OA autophagy. Co-cited authors are two or more authors simultaneously cited by another paper (18). Among 673 co-cited authors in this study, six had a citation frequency of over 100, and Caramés B was the most co-cited author (n=302), followed by Zhang Y (n=156) and Loeser RF (n=151). Among the co-cited authors, Aigner T had the highest centrality (n=45, centrality=0.16), followed by Blanco FJ (n=78, centrality=0.10) and Almonte-Becerril M (n=62, centrality=0.10).

### 3.4 Journals and cited journals

The included 732 articles were published in 160 different journals. As shown in **Table S2**, the highest number of outputs on autophagy in OA were published in *Osteoarthritis and Cartilage* (n=45, 6.15%), followed by *International Journal Of Molecular Sciences* (n=25, 3.42%) and *Cell Death & Disease* (n=15, 2.05%). Co-cited journals are two or more journals that are cited at the same time. Among the 539 co-cited journals, five were cited more than 300 times. *Osteoarthritis and Cartilage* was the top co-cited journal (n=487), followed by *Arthritis Rheum-us* (n=414), and *Annals of Rheumatic Diseases* (n=373).

### 3.5 Keyword hotspots

**Figure 4:**
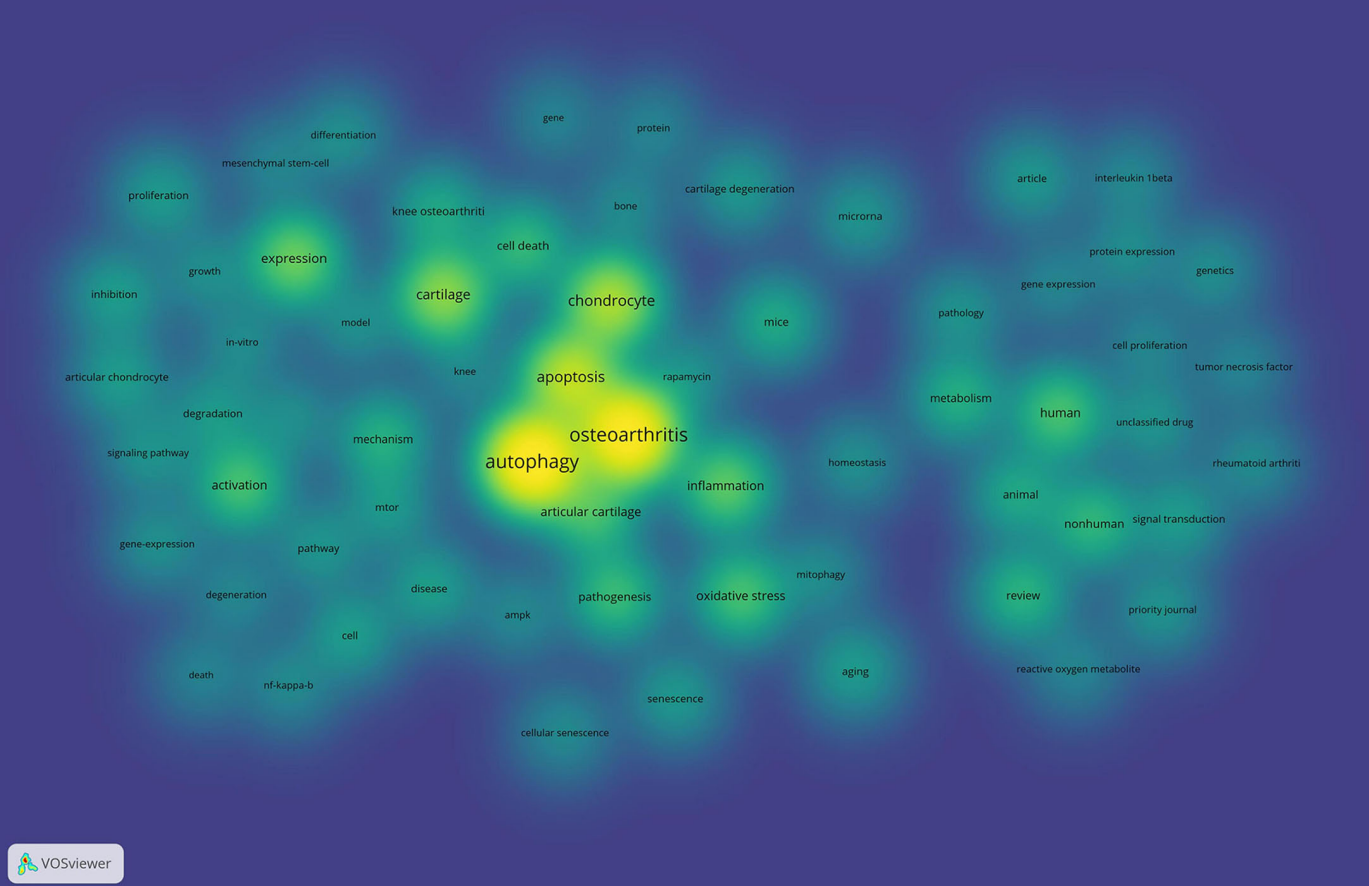
Density map of keywords related to autophagy in OA from: VOSviewer.

Keywords are often used in publications to summarize the research topic, and their analysis can reveal research hotspots and directions in a specific field. The keywords (n≥70) related to autophagy in OA are shown in **Table S3**. Among these keywords, autophagy occurred most frequently (n=296), followed by OA (n=263), apoptosis (n=189), cartilage (n=169), expression (n=153), and chondrocyte (n=123). Animal (n=66, centrality=0.21), aging (n=26, centrality=0.12), and non-human (n=86, centrality=0.10) had a centrality above 0.10, indicating the importance of these keywords in the field of autophagy in OA. **Figure 4** presents the high-frequency keywords on a density map; the intensity of the color is proportional to how frequently the keyword appears in the publication.

**Figure 5:**
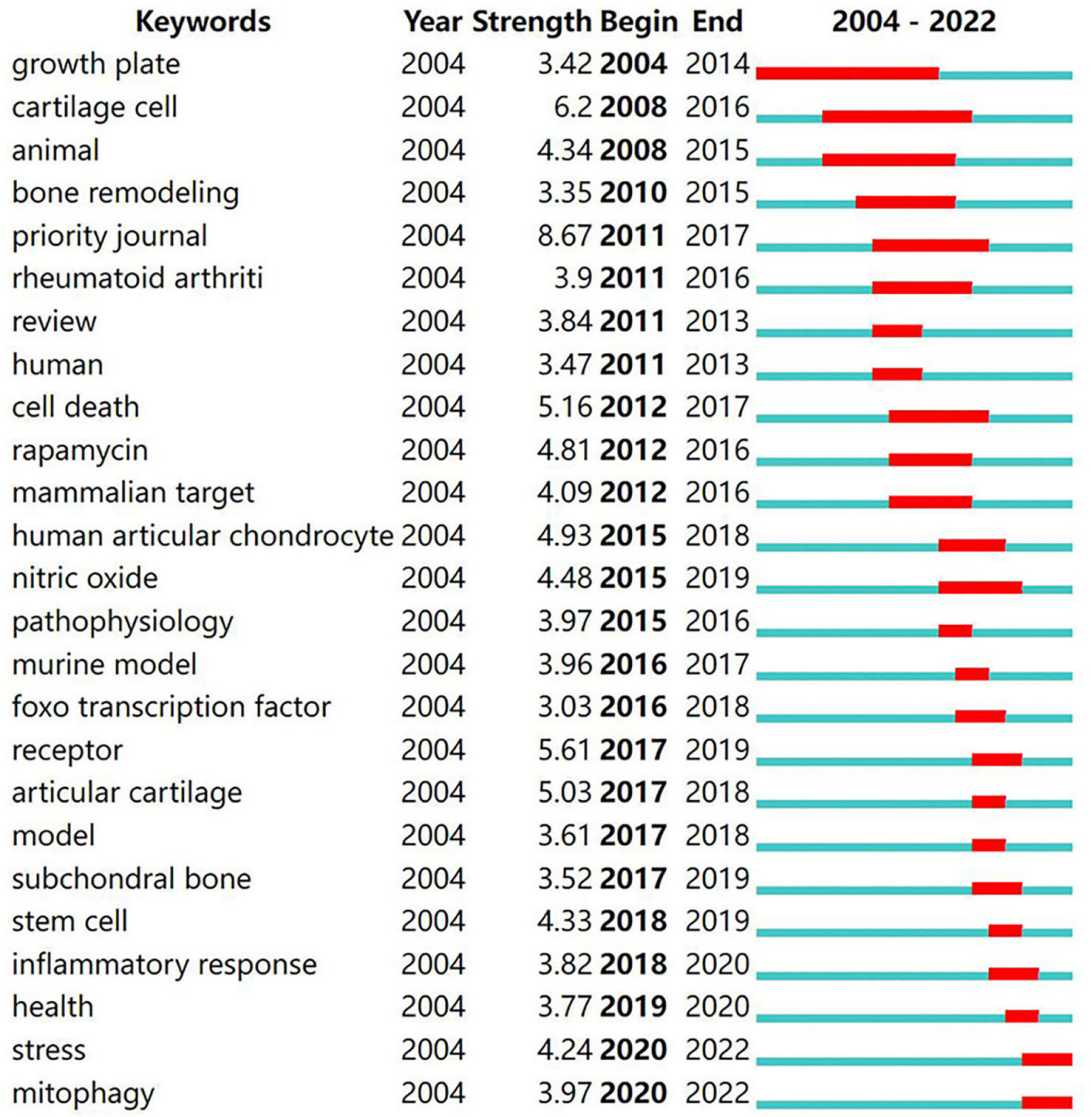
Top 25 keywords with the strongest citation bursts.

Keywords with a strong citation burst are keywords that were cited frequently over a given period. Thus, keyword citation bursts can help track the rise or fall of research hotspots. The blue line indicates the time interval, and the red line shows the time of keywords outbreak from start to completion. As shown in **Figure 5**, keywords with citation bursts first appeared in 2004 (growth plate) and persisted for a decade. “Cartilage cell” had a strong burst (strength=6.20), followed by “receptor” (strength=5.61) and “cell death” (strength=5.16). The most recent keywords with citation burst were “stress” and “mitophagy”, which appeared in 2020. The changes in keyword citation bursts over time reflect changes in the research trends.

**Figure 6:**
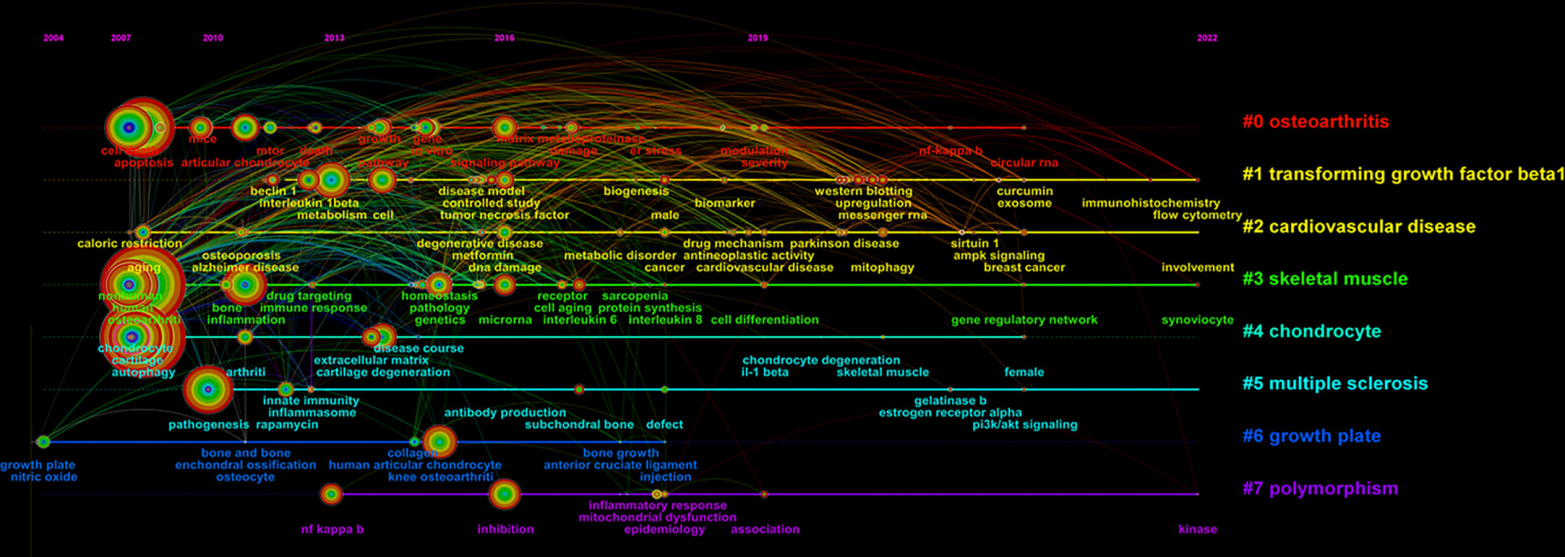
CiteSpace visualization map of timeline viewer related to autophagy in OA.

The timeline mapping of keywords is used to present the high-frequency keywords in each cluster over time. As shown in **Figure 6**, keywords on autophagy in OA were divided into the following eight clusters: OA, transforming growth factor beta 1 (TGF-β1), cardiovascular disease, skeletal muscle, chondrocyte, multiple sclerosis, growth plate, and polymorphism. Between 2004 and 2008, research on the role of autophagy in articular cartilage maintenance was in its infancy. Research during this period focused on autophagic pathways in growth plate cartilage. Since 2008, keywords such as OA, cardiovascular disease, skeletal muscle, and chondrocyte have entered the spotlight. Of these, “cardiovascular disease” has been a major research focus for some time. “Multiple sclerosis” and “TGF-β1” appeared in 2010 and 2012, respectively, and “polymorphism” began to gather attention in 2013; all three research topics have continued to be studied in recent years.

### 3.6 The research frontiers of autophagy in OA

Co-cited papers refer to two papers that are cited as references by another identical paper, and co-citation analysis can be used to track the evolution of a particular field. Of the total 680 articles cited in papers on autophagy in OA, nine were referenced more than 40 times (**Table S4**). The most cited paper was *“Cartilage-specific deletion of mTOR upregulates autophagy and protects mice from osteoarthritis”* (19). Strong reference citation bursts can be indicative of changes in research hotspots over a given time period. **Figure S1** shows the top 25 references with the strongest citation bursts involved in OA autophagy. The first burst of co-cited references appeared in 2010. Most of the co-cited references appeared in the last 10 years, implying that research related to OA autophagy may continue to advance in the future.

**Figure 7:**
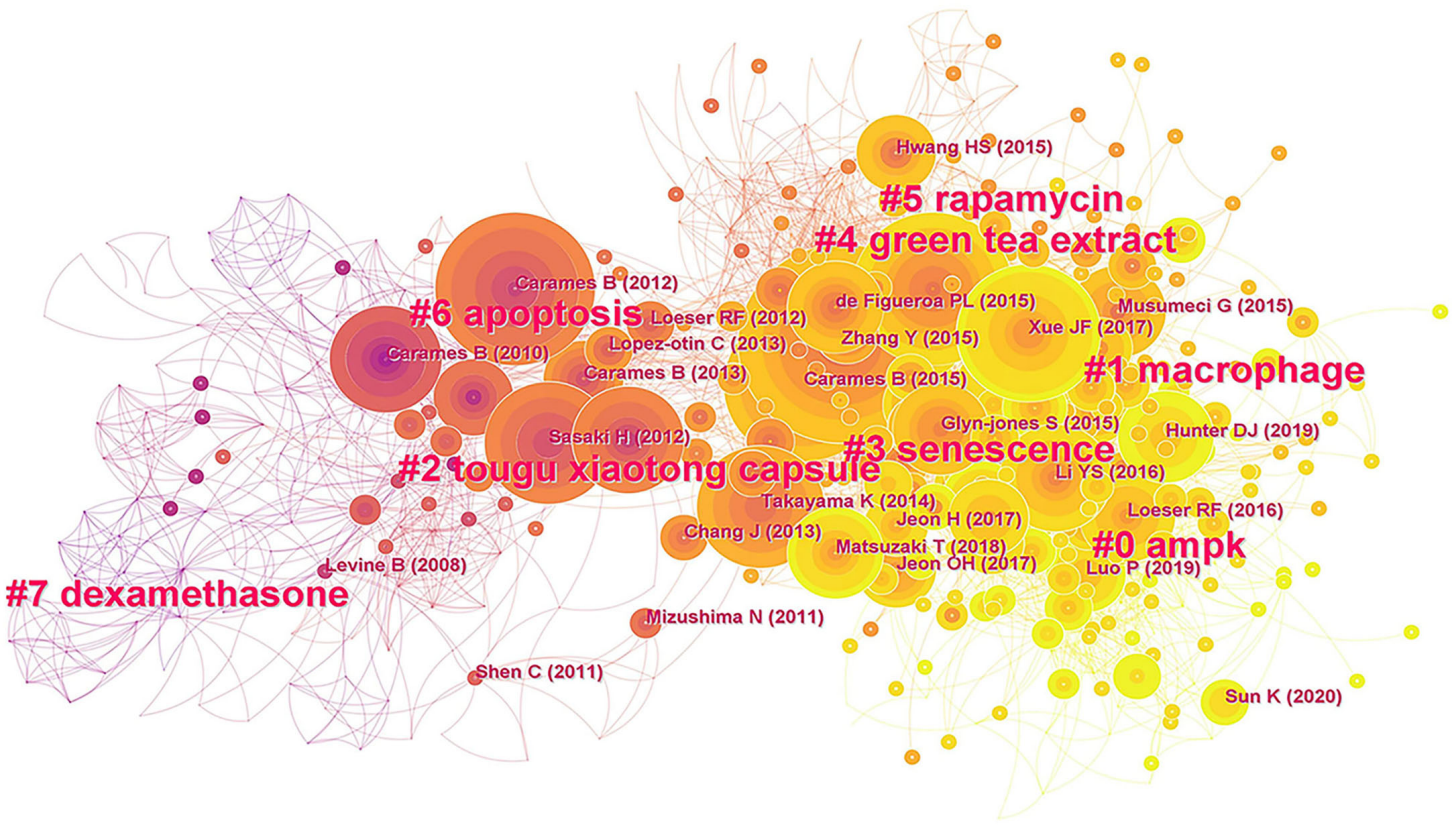
CiteSpace visualization map of co-cited references in OA autophagy.

According to the co-citation analysis, we used CiteSpace to perform a cluster analysis to extract cluster labels from the published papers and identify research frontiers in the field of OA autophagy. Credibility of clustering can be reflected by the silhouette score. A silhouette score >0.7 indicates a robust clustering result. As shown in **Table S5** and **Figure 7**, the reference network in the field of OA autophagy was extracted into eight co-citation clusters. The largest cluster was labeled “AMPK” and had 109 members with a silhouette value of 0.797. The most relevant citation for this cluster was “osteoarthritis” (n=42), published in *The Lancet* by Hunter DJ in 2019 (6). The next cluster labeled “macrophagy” had 76 members with a silhouette value of 0.698. The third cluster was labeled “tougu xiaotong capsule (TXC)” and had 61 members, with a silhouette value of 0.925. This cluster contained many papers, such as those by Caramés B (2012) and Sasaki H (2012), with strong citation bursts. Thus, AMPK, macrophage, TXC are the emerging research trends in the field of OA autophagy.

## 4 Discussion

### 4.1 General information

To the best of our knowledge, this is the first bibliometric analysis of autophagy in OA. In this study, we have identified the global hotspots and trends in this field of research. Our results show a steady increase in the cumulative number of publications in the field of OA autophagy from 2004 to 2022. The number of annual publications grew gradually between 2004 and 2017. However, since 2017, this growth has accelerated, reaching its current peak in 2021. These findings suggest that research on autophagy in OA is flourishing.

We found that China ranked top in terms of the output volume on OA autophagy, prior to the USA, South Korea, Japan, and Spain. Meanwhile, the centrality of China and the USA was far ahead of that of other countries, indicating that these countries were leading the international research efforts in the field of OA autophagy. Nine of the top 10 publishing institutions were from China, but showed a low level of collaboration. Therefore, collaboration between research institutions studying OA autophagy should be strengthened in the future. Martin Lotz and *Osteoarthritis and Cartilage* were the highest output author and journal, respectively. Meanwhile, the record for the most commonly cited papers by an author and journal were held by Caramés B and *Osteoarthritis and Cartilage*, respectively. Martin Lotz and Beatriz Caramés were among the top 10 authors and co-cited authors, reflecting their significant contributions to the field of OA autophagy. *Osteoarthritis and Cartilage* ranked first in both publications and citations, demonstrating its authority in the field. The manuscript entitled *“Cartilage-specific deletion of mTOR upregulates autophagy and protects mice from osteoarthritis”* (19), published in 2015, was most cited, and the paper entitled “*Targeted deletion of Atg5 in chondrocytes promotes age-related osteoarthritis”*, published in 2016, has the highest centrality (>0.10) (20).

### 4.2 Hotspots and frontiers in OA autophagy research

#### 4.2.1 The mechanisms of autophagy in OA

**Figure 8:**
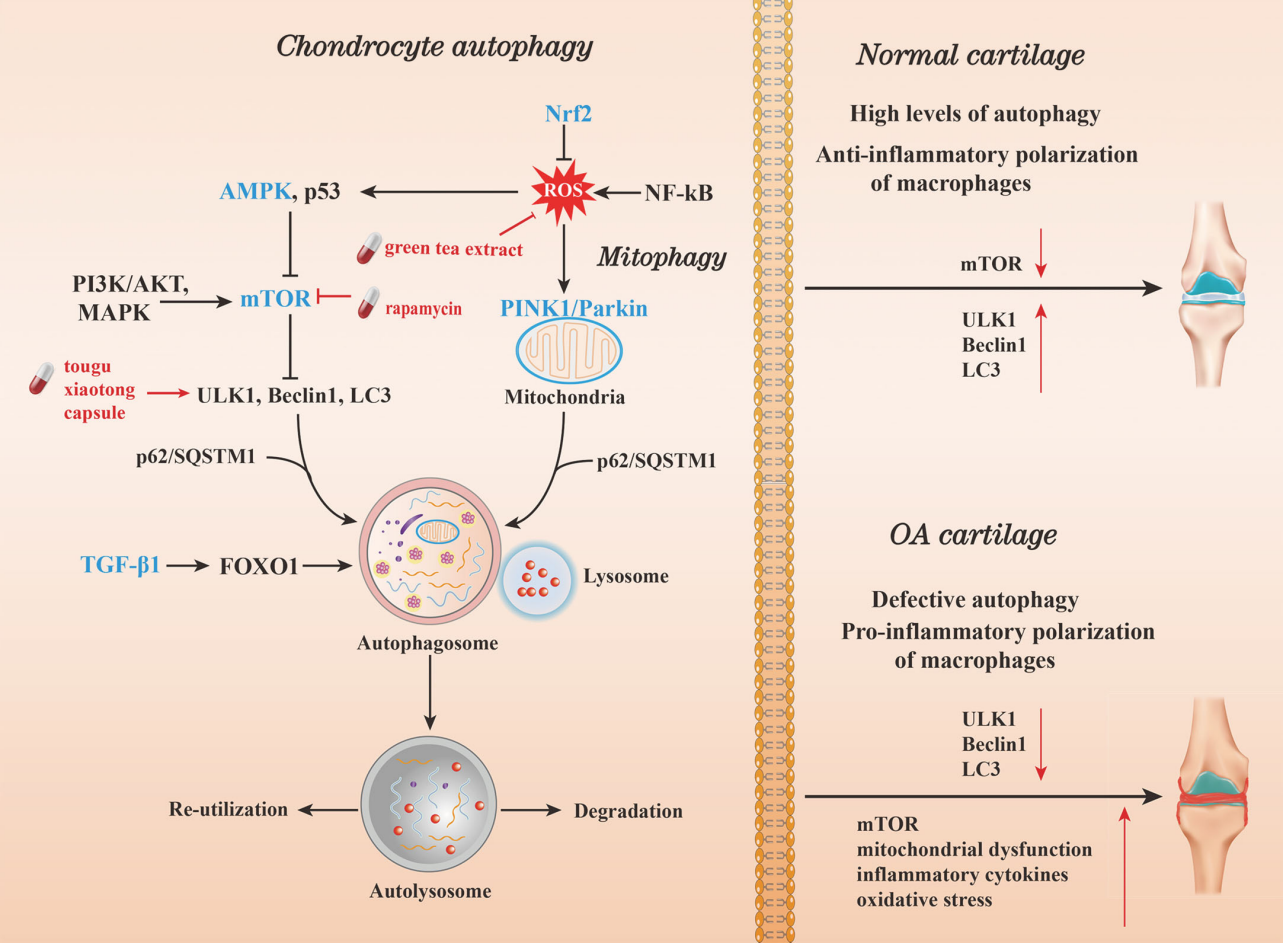
Current hotspots in autophagy pathway research in OA. Autophagy can be induced by oxidative stress. The kinase mTOR is a critical regulator of autophagy. Activation of mTOR (and consequently AKT and MAPK signaling) can inhibit autophagy. In contrast, negative regulation of mTOR (and thus, AMPK and p53 signaling) will promote autophagy. On inhibition of mTOR, downstream ULK1, Beclin1, phosphatidylinositol 3-kinase complex type 3 (PIK3C3), and LC3-II complexes, act together to promote autophagy and control autophagosome formation. While in mitophagy, the process is formed by the recruitment of the p62/SQSTM1 by the PINK1 to bind to LC3. After the autophagosome matures, it fuses with the lysosome to produce an autolysosome. The enclosed cargo is then degraded, while the nutrients and metabolites are released and re-utilized. In normal cartilage, the expression of ULK, Beclin1, and LC3 is increased, while that of mTOR is inhibited. High levels of autophagy and the polarization of anti-inflammatory M2 macrophages act as cell survival mechanisms. In OA cartilage, the expression of ULK, Beclin1, and LC3 is decreased. Consequently, autophagy is attenuated due to a drop in mTOR inhibiting factor levels, resulting in mitochondrial dysfunction, oxidative stress, inflammatory cytokine production.

OA is characterized by chondrocyte apoptosis and loss of extracellular matrix (ECM) degradation (21). For the mechanism triggering chondrocyte apoptosis, modern medical research mostly suggests that it is related to inflammation and excessive autophagy of chondrocytes (22). Current research hotspots in the study of the mechanisms of OA autophagy include inflammatory response, stress, and mitophagy. Moreover, AMP-activated protein kinase (AMPK), macrophage, and TGF-β1 are major research frontiers. Figuring out the mechanisms of autophagy in OA will help develop potential therapeutic agents and alleviate disease. Current hotspots relating to the autophagy pathway in OA are summarized in **Figure 8**.

Inflammasomes are inflammatory multiprotein complexes that can be assembled and activated in response to reactive oxygen species (ROS) or lysosomal destabilization, and are involved in inflammatory-based OA (23). The initiation of inflammasomes leads to the production of inflammatory cytokines, and stimulates increased release of cartilage degradation enzymes such as matrix metalloproteinase (MMP), causing ECM degradation (24). Excess degradation products are released into the synovial fluid, and become inflammatory mediators presented to T lymphocytes. Activated T lymphocytes invade the synovial cavity, and stimulate synovial cells to enter an inflammatory state, releasing large amounts of inflammatory factors, causing excessive autophagy, leading to deterioration of the metabolic environment, and exacerbating chondrocyte apoptosis (25). Another link between inflammasomes and OA is the association of OA with metabolic disorders such as obesity and metabolic syndrome. It has been shown that obese people have greater activity of MMP compared to those with normal weight, suggesting that the obese organism is in a chronic low-grade inflammatory state (26). Based on the ability of inflammasomes to propagate inflammation in a variety of autoimmune and auto-inflammatory conditions, inflammasome-targeted therapeutic regimens may offer a promising strategy for addressing OA.

Mitophagy had emerged as a research hotpot in recent years. Mitophagy involves targeting damaged mitochondria for degradation through receptor mediated mechanisms and plays a key role in maintaining mitochondrial homeostasis (27). Mitophagy is exacerbated by excessive catabolism, the inflammatory response, and chondrocyte death. It maintains tissue homeostasis by: 1) reducing intracellular ROS production by damaged mitochondria; 2) conserving energy by limiting the energy requirements of certain organelles; and 3) producing adenosine triphosphate (ATP) during the degradation of damaged mitochondria under physiological and pathological conditions *via* the PTEN-induced putative kinase 1 (PINK1) and Parkin pathways (28, 29). Disorders such as OA occur as a result of mitophagy perturbations. Hence, restoring mitochondrial function in chondrocytes may also be a potential therapeutic target in OA treatment (30).

AMPK plays a key role in promoting autophagy to maintain cell structure and function, *via* the inhibition of the mammalian target of rapamycin (mTOR) (31). AMPK is involved in multiple aging-related diseases, including OA (32–34). The role of AMPK in OA has been described since 2010. A reduction in AMPK activity can trigger adverse events such as mitochondrial dysfunction, oxidative stress, and impaired autophagy, which ultimately result in articular cartilage degeneration (35). Conversely, restoring AMPK activation has been demonstrated to coordinate chondrocyte survival and improve resistance to risk factors of OA (36). These findings suggest that AMPK and its associated signaling molecules may serve as potential drug targets for the treatment of OA.

Macrophages are main immune cell type within the synovial joint. They can trigger the initial inflammatory response and initiate the pathological process of OA. Macrophages can dynamically maintain homeostasis within the intra-articular microenvironment by shifting between pro-inflammatory (M1) and anti-inflammatory (M2) phenotypes (37). The transition between macrophage phenotypes is regulated by nuclear factor erythroid 2-related factor 2 (Nrf2), nuclear factor-κB (NF-κB), TGF-β, activates protein kinase B (AKT), and mitogen-activated protein kinase (MAPK) signaling (38). In obesity, adipose tissue macrophages differentiate mainly into M1 phenotypes, and produce pro-inflammatory cytokines such as IL-1β, TNF-α, and IL-6, which may be considered an important factor in OA progression (39). Targeting signaling pathways that intersect macrophage reprogramming and autophagy could provide a promising perspective for OA therapy. TGF-β1 is a critical regulator of chondrocyte homeostasis. It promotes autophagy under oxidative stress *via* Forkhead box O1 (FOXO1), which can induce the expression of autophagy-related gene (Atg) (40). In OA, the decreased expression of FOXO1 causes autophagic dysfunction in chondrocytes (41). Thus, TGF-β1 has been identified as a key regulator of articular cartilage autophagy and is another potentially therapeutic target for OA.

#### 4.2.2 Role of autophagy in OA

In the cartilage, autophagy is considered a protective mechanism for maintaining homeostasis and a normal cellular response to different types of stress. Current evidence suggests that in OA, chondrocytes undergo a decline in the expression of autophagy markers, including the autophagy activating kinase 1 (ULK1), bcl-2 interacting coiled-coil protein (Beclin1), Atg5, and light chain 3 (LC3), and an increase in apoptosis (42). In this study, we identified the crosstalk between autophagy, apoptosis, and senescence as both a research hotspot and frontier. It has been demonstrated that cell death in OA chondrocytes occurs *via* a combination of classical apoptosis and autophagy (43). In the early stages of OA, autophagy is activated as an adaptive survival response, which results in increased autophagic marker expression on the chondrocytes. However, in the later stages of OA, when the chondrocytes become severely damaged, autophagy is activated together with apoptosis as an alternative pathway for evading cell death and potentially inducing senescence. These findings suggest that the role of autophagy is different in distinct stages of OA, and that potential correlations exist between OA severity, apoptosis, and autophagy. Additional studies are needed to further explore the role of autophagy in distinct stages of OA, so as to better prevent the OA progression.

#### 4.2.3 Application of autophagy as a therapeutic tool in OA

In this study, we identified “dexamethasone” and “rapamycin” as research frontiers in the field of OA autophagy. Findings from *in vitro* experiments showed that dexamethasone can induce protective autophagy in chondrocytes in the early stage of OA, and that this effect was gradually attenuated over time (44). However, it has been reported that prolonged dexamethasone use may accelerate OA progression by decreasing autophagy and promoting apoptosis. Moreover, the safety of dexamethasone has been questioned, as it could induce cardiovascular events in patients (45). At present, only the intra-articular injection of dexamethasone is conditionally recommended as a short-term pain reliever for some patients with of OA with persistent or moderate to severe pain (46). Rapamycin, which is commonly used in experimental models of OA, induces autophagy by inhibiting mTOR hyperactivation (47). Rapamycin is currently used to prevent organ rejection following transplantation and to treat certain types of cancer. However, its long-term application is limited by potential adverse effects, such as blood and metabolic disorders (48). Trials of rapamycin as an OA therapy are still in the preclinical stage. Further high-quality studies of the efficacy and safety of rapamycin analogs are warranted to support the use of mTOR inhibitors in treating OA. Currently, intra-articular stem cell injections have been approved for clinical trials in treating knee OA. A controlled, double-blind clinical trial showed that an intra-articular injection of bone marrow-derived mesenchymal stem cells with or without the addition of platelet-rich plasma displayed the highest percentage of improvement in most Knee Injury and Osteoarthritis Outcome Score domains and global score compared to corticosteroid injections, effectively improving function and reducing symptoms caused by knee OA (49). However, its efficacy and safety still need to be observed in long-term clinical trials.

Due to the side effects and clinical uncertainties of mainstream medicine, complementary and alternative medicine (CAM) gradually gained attention. To date, CAM such as herbal medicine, acupuncture, massage therapy, and nutritional supplements has become popular in patients with OA worldwide (50). As the amazing part of traditional Chinese medicine (TCM), herbal medicine has been used in China over 2,500 years for OA. Current studies demonstrated that herbal medicine contains ample bioactive substances, some of which have been suggested to have antioxidant or anti-inflammatory activity and are highly correlated with autophagy (51). Our results showed that among autophagy promoting agents, “TXC” and “green tea extract” are research frontiers in the field of OA autophagy.

TXC is a TCM, which contains four natural ingredients, including Morindae officinalis, Radix Paeoniae Alba, Rhizoma Chuanxiong, and Sarcandra glabra. These components have demonstrated potential therapeutic effects on OA. Monotropein, an iridoids glycoside isolated from the roots of Morindae officinalis, has been reported to have anti-inflammatory and analgesic, antioxidant, and osteoprotective effect (52). As one of the major active components of Radix Paeoniae Alba, paeoniflorin is the first botanical anti-rheumatic drug with anti-inflammatory immunomodulatory properties by modulating the anti-inflammatory phenotype of macrophages (53). Ferulic acid, one of the active ingredients of Rhizoma Chuanxiong, has pharmacological activities in scavenging of oxygen free radicals and reducing oxidative stress (54). As for isofraxidin, the characteristic compound of coumarins in Sarcandra glabra, has also been verified to reduce the serum levels of inflammatory cytokines (55). To some extent, the synergistic effect of these bioactive substances determines the therapeutic properties of TXC in up-regulating autophagic activity. TXC appears to enhance chondrocyte autophagy by promoting the expression of LC3, ULK1, and Beclin1 (56). TXC was also shown to regulate the LC3 conjugation system to inhibit cartilage degradation in OA (57).

Apart from TXC, a variety of herbal compound formulas have been used clinically for the treatment of OA in China, which may be associated with its chondroprotective effects through regulating autophagy of chondrocytes (58). The results of multicenter randomized controlled trials (RCT) showed that OA patients treated with shaoyang xibi decoction had lower Western Ontario and McMaster Universities Osteoarthritis Index (WOMAC) score and less adverse events than those treated with meloxicam alone (59). Another RCT showed that clearing heat and dispelling paralysis soup can better improve motor ability, and reduce the development of inflammation in the organism, with high safety and effectiveness compared with loxoprofen sodium dispersible tablets (60). A pooled analysis showed that duhuo jisheng decoction can reduce the pain and WOMAC scores in the treatment of Knee OA (61). A meta-analysis showed that NSAIDs combined with jinwu gutong capsule can further improve the clinical efficacy of Knee OA (62). Further high-quality evidence is still needed to support the application of TCM for OA, and more efforts are needed to elucidate the mechanisms of TCM in treating OA.

Among the different forms of nutritional supplements, green tea is favored for its multiple beneficial effects on the human body. Polyphenols, the major bioactive components of green tea, may regulate autophagy by down-regulating inflammatory signaling mediators, up-regulating anabolic mediators, and modulating microRNA expression. If left unchecked, these processes lead to increased chondrocyte death, collagen degradation, and eventually OA progression (63). Additional clinical evidence is needed to assess the effects of green tea extract in patients with OA. Besides green tea extract, many natural plant components such as quercetin and resveratrol can also alleviate articular cartilage degeneration by activating AMPK signaling, reducing oxidative stress and mitochondrial dysfunction in chondrocytes (31). Moreover, resveratrol can delay OA progression by activating Sirtuin 1 protein (64). Herbal extracts such as curcumin and icariin can regulate OA chondrocyte autophagy and reduce chondrocyte apoptosis by regulating the PI3K/Akt/mTOR signaling pathway (65, 66); while dihydroartemisinin and sinomenine can activate autophagy by inhibiting the NF-κB pathway (67, 68). These candidates show potential in the adjunctive treatment of OA, but more efforts are needed to support their use.

Worth mentioning, progress in the field of OA autophagy has led to the development of novel targeted drugs with therapeutic potential. For example, AMPK activators coordinate chondrocyte metabolism, Nrf2 mediates anti-inflammatory polarization of synovial macrophages, TGF-β superfamily members increase the synthesis of cartilage ECM, zinc facilitates the PINK1-dependent mitophagy pathway in OA chondrocytes, and specific miRNAs regulate the epigenetic mechanisms of autophagy (69). Nevertheless, most of these candidates are still in preclinical stage and more efforts are needed to support their use in OA treatment. In summary, the development of new drugs that enhance or restore autophagic activity is a promising strategy for the treatment of OA.

### 4.3 Limitations and prospects in OA autophagy research

Autophagy has already been awarded the Nobel prize. Prior studies of OA autophagy mainly focused on mechanisms underlying OA and autophagy, usually involving the characterization and localization of specific subcellular forms (e.g., autophagosome and autolysosome) at the cellular level, and the quantification of autophagy-specific proteins (e.g., LC3-II) at the molecular level (70). Our findings revealed that selective autophagy in OA has received increasing attention, with a focus on mitophagy and er-phagy, and substantial progress has been made in elucidating its regulatory mechanism. The reason for the selectivity is that certain autophagy-related proteins or other effectors to specifically recognize receptor molecules on mitochondria or endoplasmic reticulum, and then initiate autophagosome formation on their surfaces (71). Therefore, in autophagy research, it is more important to focus on its selective features, such as the observation and analysis of co-localization of autophagosomes with specific organelles, and the detection and analysis of expression levels and modification levels of characteristic linker molecules related to the mechanism (72). With the advancement of modern biological technology, the integration of traditional detection methods and emerging technologies can further improve the autophagy research system.

Given that autophagy is a molecular-level regulatory mechanism with subcellular morphological characteristics in the cellular microenvironment, the study of autophagy is easier to achieve at the cellular level *in vitro*, while the technical requirements for the *in vivo* study of autophagy at the holistic level are relatively high. Atgs are closely related to cell survival. Knockdown of Atgs such as Beclin1, often leads to the death of experimental animals during the embryonic or neonatal stages, so *in vivo* studies of autophagy at the overall level are currently only possible through partial knockdown of genes or knockdown of autophagy genes in specific organs (73, 74). The limited *in vivo* experiments have to some extent prevented autophagy target drugs from entering the clinical research stage. In future studies, there is a need to search for more reliable biomarkers of OA autophagy including new selective autophagy receptors, kinases and other effectors, develop new *in vivo* research techniques, and make new progress in autophagy study in OA.

The pathogenesis of OA is complex and closely related to autophagy, senescence, and apoptosis. Autophagy as an adaptive response can reduce chondrocyte death in the early stages of OA, but excessive autophagy may also lead to chondrocyte death as OA progresses (75). There are still many unproven mechanisms in OA autophagy research, such as the mechanism of synergistic regulation and intrinsic connection between mitophagy and er-phagy in OA, and the regulation of autophagy levels in pathological states. All these need to be further explored in future.

Autophagy has a broad clinical prospect in the treatment of OA, and targeting autophagic pathways provides a new direction for OA treatment. Particularly, locally targeted drug delivery, such as intra-articular injection of autophagy-regulated drugs, is expected to become a new hotspot in the treatment of OA. In the future, clinical studies on the treatment of OA with autophagy-regulated drugs can be carried out gradually. With the support of high quality clinical evidence, autophagy-activating drugs may be used to enhance or restore autophagic activity, thereby to stop disease progression for patients with early-stage OA; for those with intermediate and advanced stages, autophagy-inhibiting drugs may be applied to reduce excessive autophagy, so as to improve pain and joint function, and delay disease progression. In addition, some natural active substances with autophagy-regulating effects may be used as adjuvant therapy to further improve the clinical efficacy of OA, which also provides a new idea for the treatment of OA.

### 4.4 Limitations

This study has some shortcomings. Firstly, we used CiteSpace software for data analysis, which may eliminate some data components from the analysis. Thus, we may have missed some detailed data. Secondly, our study was based on literature published between 2004 and 2022, meaning that our findings are only relevant to this research period and will need to be updated to keep pace with the volume of new research outputs.

## 5 Conclusion

Research on autophagy in the field of OA is flourishing. China exhibited the highest output volume, prior to the USA, and South Korea. Martin Lotz and Beatriz Caramés are outstanding contributors to this field. *Osteoarthritis and Cartilage* is the most active and popular journal. Past research of autophagy in OA mainly focused on the mechanistic aspects such as the roles of AMPK, macrophages, TGF-β1, inflammatory response, stress, and mitophagy. However, emerging research trends include: 1) delineating the relationship between autophagy, apoptosis, and senescence; and 2) testing the efficacy of therapeutic agents such as TXC and green tea extract in OA treatment. New targeted drugs are under development, but most are still in the preclinical stage. We envisage that advances in autophagy research accompanied by the emergence of targeted drugs will one day lead to the development of precision medicine for OA.

## Data availability statement

The original contributions presented in the study are included in the article/**Supplementary Material**. Further inquiries can be directed to the corresponding authors.

## Author contributions

JL and QWT contributed to conception and design of the work. JHL, XBY developed the search strategy. JHL, QH, YZ and WJS performed the data acquisition, analysis, and interpretation. JHL wrote the original draft preparation. JL, QWT, XBY, JQC, and ZHW provided the critical revision of the draft. All authors give approval of the final version to be published and take full responsibility for the integrity and accuracy of all aspects of the work.
