# Supplementary material for: Knowledge mapping of autophagy in osteoarthritis from 2004 to 2022: A bibliometric analysis
Source: Front Immunol. 2023 Mar 9;14:1063018. doi: 10.3389/fimmu.2023.1063018 (PMC10033547; doi:10.3389/fimmu.2023.1063018)
Supplement: Supplementary file 1 [file DataSheet_1.zip › Supplementary Material/Supplementary Figure.pdf]

Supplementary Figure

References	Year	Strength	Begin	End	2004 - 2022
Carames B, 2010, ARTHRITIS RHEUM-US, V62, P791, DOI 10.1002/art.27305, DOI	2010	20.83	**2010**	2015	
Bohensky J, 2009, ARTHRITIS RHEUM-US, V60, P1406, DOI 10.1002/art.24444, DOI	2009	7.09	**2010**	2014	
Almonte-becerril M, 2010, APOPTOSIS, V15, P631, DOI 10.1007/s10495-010-0458-z, DOI	2010	6.97	**2011**	2015	
Bohensky J, 2010, PEDIATR NEPHROL, V25, P633, DOI 10.1007/s00467-009-1310-y, DOI	2010	5.36	**2011**	2015	
Lotz MK, 2011, NAT REV RHEUMATOL, V7, P579, DOI 10.1038/nrrheum.2011.109, DOI	2011	12.94	**2012**	2016	
Carames B, 2012, ANN RHEUM DIS, V71, P575, DOI 10.1136/annrheumdis-2011-200557, DOI	2012	26.61	**2013**	2017	
Sasaki H, 2012, ARTHRITIS RHEUM-US, V64, P1920, DOI 10.1002/art.34323, DOI	2012	21.66	**2013**	2017	
Carames B, 2012, ARTHRITIS RHEUM-US, V64, P1182, DOI 10.1002/art.33444, DOI	2012	11.52	**2013**	2017	
Carames B, 2013, ARTHRITIS RHEUM-US, V65, P1843, DOI 10.1002/art.37977, DOI	2013	7.96	**2014**	2018	
Loeser RF, 2012, ARTHRITIS RHEUM-US, V64, P1697, DOI 10.1002/art.34453, DOI	2012	6.32	**2015**	2017	
Chang J, 2013, INT J MOL MED, V32, P1311, DOI 10.3892/ijmm.2013.1520, DOI	2013	5.11	**2015**	2018	
Takayama K, 2014, ARTHRITIS RES THER, V16, P0, DOI 10.1186/s13075-014-0482-4, DOI	2014	14.19	**2016**	2019	
Zhang Y, 2015, ANN RHEUM DIS, V74, P1432, DOI 10.1136/annrheumdis-2013-204599, DOI	2015	10.27	**2016**	2019	
Carames B, 2015, ARTHRITIS RHEUMATOL, V67, P1568, DOI 10.1002/art.39073, DOI	2015	9.84	**2016**	2019	
Matsuzaki T, 2014, BIOMATERIALS, V35, P9904, DOI 10.1016/j.biomaterials.2014.08.041, DOI	2014	5.65	**2016**	2018	
Akasaki Y, 2014, ARTHRITIS RHEUMATOL, V66, P3349, DOI 10.1002/art.38868, DOI	2014	4.84	**2016**	2018	
Shapiro IM, 2014, AUTOPHAGY, V10, P7, DOI 10.4161/auto.26679, DOI	2014	4.84	**2016**	2018	
Musumeci G, 2015, INT J MOL SCI, V16, P20560, DOI 10.3390/ijms160920560, DOI	2015	5.54	**2018**	2020	
Glyn-jones S, 2015, LANCET, V386, P376, DOI 10.1016/S0140-6736(14)60802-3, DOI	2015	5.1	**2018**	2020	
Hwang HS, 2015, INT J MOL SCI, V16, P26035, DOI 10.3390/ijms161125943, DOI	2015	6.44	**2019**	2020	
Jeon H, 2017, CONNECT TISSUE RES, V58, P497, DOI 10.1080/03008207.2016.1240790, DOI	2017	5.04	**2019**	2022	
Luo P, 2019, BIOMED RES INT, V2019, P0, DOI 10.1155/2019/5171602, DOI	2019	8.11	**2020**	2022	
Xue JF, 2017, BIOMED PHARMACOTHER, V89, P1252, DOI 10.1016/j.biopha.2017.01.130, DOI	2017	6.17	**2020**	2022	
Loeser RF, 2016, NAT REV RHEUMATOL, V12, P412, DOI 10.1038/nrrheum.2016.65, DOI	2016	5.57	**2020**	2022	
Ansari MY, 2018, OSTEOARTHR CARTILAGE, V26, P1087, DOI 10.1016/j.joca.2017.07.020, DOI	2018	5.33	**2020**	2022	

Supplementary Figure 1. Top 25 references with the strongest citation bursts.
