# Supplementary material for: Knowledge mapping of autophagy in osteoarthritis from 2004 to 2022: A bibliometric analysis
Source: Front Immunol. 2023 Mar 9;14:1063018. doi: 10.3389/fimmu.2023.1063018 (PMC10033547; doi:10.3389/fimmu.2023.1063018)
Supplement: Supplementary file 1 [file DataSheet_1.zip › Supplementary Material/Supplementary Tables.docx]

***Supplementary Tables***

**Supplementary Table 1.** Top authors (n≥5) and co-cited authors (n≥80) related to autophagy in OA.

| **Rank** | **Author** | **Year** | **Centrality** | **Count (%)** | **Co-Cited Author** | **Year** | **Centrality** | **Citation** |
| --- | --- | --- | --- | --- | --- | --- | --- | --- |
| 1 | Martin Lotz | 2010 | 0.01 | 30 (4.10) | Carames B | 2010 | 0.10 | 302 |
| 2 | Beatriz Caramés | 2011 | ＜0.01 | 13 (1.78) | Zhang Y | 2015 | 0.02 | 156 |
| 3 | Bai Lunhao | 2017 | ＜0.01 | 10 (1.37) | Loeser RF | 2011 | 0.06 | 151 |
| 4 | Zhang Xiaolei | 2018 | ＜0.01 | 6 (0.82) | Mizushima N | 2010 | 0.04 | 146 |
| 5 | Chen Lin | 2019 | ＜0.01 | 5 (0.68) | Sasaki H | 2013 | 0.03 | 129 |
| 6 | Wu Lidong | 2020 | ＜0.01 | 5 (0.68) | Lotz MK | 2012 | 0.05 | 124 |
| 7 | Wu Yaosen | 2019 | ＜0.01 | 5 (0.68) | Goldring MB | 2011 | 0.06 | 94 |
| 8 | Zhang Jian | 2016 | ＜0.01 | 5 (0.68) | Levine B | 2010 | 0.05 | 90 |
| 9 | Zhang Rui | 2019 | ＜0.01 | 5 (0.68) | Glasson SS | 2014 | 0.03 | 84 |

**Supplementary Table 2.** Top 10 journal and co-cited journals related to autophagy in OA.

| **Rank** | **Journal** | **Count (%)** | **Co-Cited Journal** | **Citation** |
| --- | --- | --- | --- | --- |
| 1 | Osteoarthritis and Cartilage | 45 (6.15) | Osteoarthritis and Cartilage | 487 |
| 2 | International Journal Of Molecular Sciences | 25 (3.42) | Arthritis Rheum-us | 414 |
| 3 | Cell Death & Disease | 15 (2.05) | Annals Of the Rheumatic Diseases | 373 |
| 4 | Frontiers In Pharmacology | 14 (1.91) | Arthritis Research & Therapy | 326 |
| 5 | International Journal Of Molecular Medicine | 13 (1.78) | Nature Reviews Rheumatology | 300 |
| 6 | Cells | 13 (1.78) | Autophagy | 286 |
| 7 | Cartilage | 12 (1.64) | Journal of Biological Chemistry | 280 |
| 8 | Arthritis Research & Therapy | 12 (1.64) | Cell | 279 |
| 9 | Arthritis & Rheumatology | 11 (1.50) | Nature | 268 |
| 10 | Life Sciences | 11 (1.50) | International Journal of Molecular Sciences | 235 |

**Supplementary Table 3.** Top Keywords (n≥70) related to autophagy in OA.

| **Rank** | **Keywords** | **Year** | **Centrality** | **Count** | **Rank** | **Keywords** | **Year** | **Centrality** | **Count** |
| --- | --- | --- | --- | --- | --- | --- | --- | --- | --- |
| 1 | autophagy | 2008 | 0.06 | 296 | 10 | articular cartilage | 2010 | 0.04 | 110 |
| 2 | osteoarthritis | 2008 | 0.04 | 263 | 11 | inflammation | 2011 | 0.03 | 100 |
| 3 | apoptosis | 2008 | 0.03 | 189 | 12 | pathogenesis | 2010 | 0.04 | 94 |
| 4 | cartilage | 2008 | 0.02 | 169 | 13 | cell death | 2008 | 0.05 | 92 |
| 5 | expression | 2009 | 0.01 | 153 | 14 | nonhuman | 2008 | 0.10 | 86 |
| 6 | chondrocyte | 2008 | 0.01 | 123 | 15 | review | 2008 | 0.01 | 82 |
| 7 | human | 2008 | 0.08 | 114 | 16 | mechanism | 2013 | 0.01 | 74 |
| 8 | oxidative stress | 2010 | 0.03 | 113 | 17 | metabolism | 2013 | 0.05 | 74 |
| 9 | activation | 2009 | 0.03 | 113 | 18 | knee osteoarthriti | 2015 | 0.01 | 72 |

**Supplementary Table 4.** Top 10 references related to autophagy in OA.

| **Rank** | **Title** | **DOI** | **Year** | **Centrality** | **Citation** |
| --- | --- | --- | --- | --- | --- |
| 1 | Cartilage-specific deletion of mTOR upregulates autophagy and protects mice from osteoarthritis | 10.1136/annrheumdis-2013-204599 | 2015 | 0.07 | 90 |
| 2 | The Relationship of Autophagy Defects to Cartilage Damage During Joint Aging in a Mouse Model | 10.1002/art.39073 | 2015 | 0.06 | 66 |
| 3 | Autophagy activation by rapamycin reduces severity of experimental osteoarthritis | 10.1136/annrheumdis-2011-200557 | 2012 | 0.03 | 66 |
| 4 | Autophagy modulates osteoarthritis-related gene expression in human chondrocytes | 10.1002/art.34323 | 2012 | 0.06 | 54 |
| 5 | Inhibition of PI3K/AKT/mTOR signaling pathway promotes autophagy of articular chondrocytes and attenuates inflammatory response in rats with osteoarthritis | 10.1016/j.biopha.2017.01.130 | 2017 | 0.04 | 51 |
| 6 | Autophagy in osteoarthritis | 10.1016/j.jbspin.2015.06.009 | 2016 | 0.01 | 48 |
| 7 | Local intra-articular injection of rapamycin delays articular cartilage degeneration in a murine model of osteoarthritis | 10.1186/s13075-014-0482-4 | 2014 | 0.03 | 46 |
| 8 | Osteoarthritis | 10.1016/S0140-6736(19)30417-9 | 2019 | 0.01 | 42 |
| 9 | Targeted deletion of Atg5 in chondrocytes promotes age-related osteoarthritis | 10.1136/annrheumdis-2015-207742 | 2016 | 0.11 | 42 |
| 10 | Ageing and the pathogenesis of osteoarthritis | 10.1038/nrrheum.2016.65 | 2016 | 0.02 | 39 |
| 11 | Autophagy Is a Protective Mechanism in Normal Cartilage, and Its Aging-Related Loss Is Linked With Cell Death and Osteoarthritis | 10.1002/art.27305 | 2010 | 0.08 | 39 |

**Supplementary Table 5.** The clusters of co-cited references in OA autophagy.

| **ClusterID** | **Size** | **Silhouette** | **Mean (Year)** | **Label** |
| --- | --- | --- | --- | --- |
| 0 | 109 | 0.797 | 2020 | ampk |
| 1 | 76 | 0.698 | 2019 | macrophagy |
| 2 | 61 | 0.925 | 2012 | tougu xiaotong capsule |
| 3 | 60 | 0.847 | 2018 | senescence |
| 4 | 59 | 0.864 | 2016 | green tea extract |
| 5 | 57 | 0.867 | 2017 | rapamycin |
| 6 | 51 | 0.847 | 2013 | apoptosis |
| 7 | 49 | 0.942 | 2011 | dexamethasone |
